# Silencing TRPM7 in Mouse Cortical Astrocytes Impairs Cell Proliferation and Migration via ERK and JNK Signaling Pathways

**DOI:** 10.1371/journal.pone.0119912

**Published:** 2015-03-23

**Authors:** Zhao Zeng, Tiandong Leng, Xuechao Feng, Huawei Sun, Koichi Inoue, Li Zhu, Zhi-Gang Xiong

**Affiliations:** 1 Cyrus Tang Hematology center, Collaborative Innovation Center of Hematology, MOH Key Lab of Thrombosis and Hemostasis, Jiangsu Key Lab of Preventive and translational Medicine for Geriatric Diseases, Soochow University, Suzhou, Jiangsu, China; 2 Neuroscience Institute, Morehouse School of Medicine, Atlanta, GA, United States of America; 3 Membrane Channel Research Laboratory and Key Laboratory for Applied Statistics of MOE, Northeast Normal University, Changchun, P.R. China; 4 Department of Cardiology, Sun Yat-sen University, Guangzhou, Guangdong, China; Massachusetts General Hospital/Harvard Medical School, UNITED STATES

## Abstract

Transient receptor potential melastatin 7 (TRPM7), a non-selective cation channel, is highly expressed expressed in the brain and plays a critical role in ischemic neuronal death. Astrocyte, the most abundant cell type in central nervous system (CNS), exerts many essential functions in the physiological and pathological conditions. Here we investigated the expression and functions of the TRPM7 channel in mouse cortical astrocytes. Using reverse transcription (RT)-PCR, immunostaining, western blot and patch clamp recording, we showed that functional TRPM7 channel is expressed in cultured mouse cortical astrocytes. Knocking down TRPM7 with specific siRNA impairs the proliferation and migration of astrocytes by 40.2% ± 3.9% and 40.1% ± 11.5%, respectively. Consistently, inhibition of TRPM7 with 2-aminoethoxydiphenyl borate (2-APB) also decreases astrocyte proliferation and migration by 46.1% ± 2.5% and 64.2% ± 2.4%. MAPKs and Akt signaling pathways have been shown to be implicated in TRPM7-mediated responses including cell proliferation and migration. Our data show that suppression of TRPM7 in astrocytes reduces the phosphorylation of extracellular signal-regulated kinases (ERK) and c-Jun N-terminal kinases (JNK), but not p38 mitogen-activated protein kinase and Akt. In addition, TRPM7, as a cation channel, has been involved in the Ca^2+^ and Mg^2+^ homeostasis in several types of cells. In our study, we found that silencing TRPM7 decreases the intracellular basal Mg^2+^ concentration without affecting Ca^2+^ concentration in astrocytes. However, an addition of Mg^2+^ to the growth medium could not rescue the impaired proliferation of astrocytes. Together, our data suggest that TRPM7 channel may play a critical role in the proliferation and migration of astrocytes via the ERK and JNK pathways.

## Introduction

Astrocytes are specialized glial cells that outnumber neurons by over five folds in CNS. They play a variety of roles such as regulating the release of neurotrophic factors, modulating neuronal development and functions, metabolizing neurotransmitters, and regulating extracellular ion level and immune response [1–3]. Astrocytes can be activated and proliferated in response to some pathophysiological factors. These processes are called reactive astrogliosis. Reactive astrogliosis has become a pathological hallmark of CNS structural lesions and a therapeutic target for neurodegenerative diseases. Based on the dysfunction of astrocytes in some pathologic status, the strategy to restore or enhance astrocyte functions could be an appealing way to promote brain functions [4]. Transient receptor potential (TRP) ion channels play an important role in diverse cellular processes in the CNS. Astrocytes express several TRP channels including TRPA1, TRPC1, TRPC3, TRPC4, TRPC5, TRPV2 and TRPV4. They play crucial roles in the regulation of astrocytic functions [5–9]. For example, TRPA1 was reported to regulate astrocyte intracellular Ca^2+^ concentration and inhibitory synaptic transmission [9, 10]. TRPV4 is involved in ischemia-mediated increases of intracellular Ca^2+^ in astrocytes [8]. TRPC3 mediates thrombin-induced astrocyte activity and upregulates its own expression [11]. However, the expression and function of TRPM7 in astrocytes has not been elucidated.

TRPM7 is a member of the melastatin-related subfamily of TRP channels [12, 13]. It is a ubiquitously expressed channel protein that conducts diverse cations including Ca^2+^ and Mg^2+^, and possesses an alpha kinase domain in its C-terminal [13–16]. Increasing evidences show that TRPM7 channel plays an important role in fundamental cellular processes including survival, proliferation, cell cycle progression, magnesium homeostasis, and responses to shear stress and oxidative stress [15–17]. Previous studies indicated that TRPM7 is highly expressed in the brain and plays a critical role in anoxic neuronal death by mediating Ca^2+^ influx during the cerebral ischemia and prolonged oxygen-glucose deprivation (OGD) [18–21]. Suppression of TRPM7 in hippocampus CA1 neurons facilitates neuron survival after brain ischemia, and preserves neuronal morphology and function [22]. Thus, TRPM7 has been considered as a potential target for treatment of ischemia brain injury [22]. In addition, TRPM7 is also involved in several neurodegenerative diseases such as western pacific amyotrophic lateral sclerosis (ALS), parkinsonism dementia (PD), and Alzheimer’s disease (AD) [23, 24]. However, the studies on the role of TRPM7 in glial cells are still rare. Jiang et al. first identified TRPM7 transcripts and TRPM7-like current in rat microglia [25]. Recently, Siddiqui et al found that TRPM7 enhances the migration and invasion of microglia in anti-inflammatory states [26]. However, the physiological functions of TRPM7 in astrocytes are largely unknown. The present study demonstrates that the functional TRPM7 channel exists in mouse cortical astrocytes. Knocking down or inhibiting TRPM7 impairs the proliferation and migration of astrocytes. Previous studies showed that TRPM7 regulates cell proliferation and migration through several signaling pathways including MAPK and PI3K/AKT pathways [27–29]. The current study demonstrated that knocking down or inhibiting TRPM7 impairs the proliferation and migration of astrocytes via the ERK and JNK, but not p38 and Akt signaling pathways. In addition, TRPM7 channel, as a cation channel, is likely involved in the regulation of intracellular Mg^2+^ concentration of astrocytes.

## Materials and Methods

### Reagents and antibodies

2-APB, propidium iodide (PI) and protease inhibitors cocktail were purchased from Sigma (St. Louis, MO). Lactate dehydrogenase (LDH) assay kit and phosphatase inhibitors cocktail were from Roche (Indianapolis, IN). Fluo-3/acetoxymethyl ester (Fluo-3/AM) was purchased from Beyotime Institute of Biotechnology (Jiangsu, China). Mouse monoclonal anti-TRPM7 antibody (Cat# ab85016) and rabbit polyclonal anti-β-actin antibody (Cat# ab8227) were purchased from Abcam (Cambridge, MA). Rabbit polyclonal antibodies against phosphorylated-Akt (p-Akt, Ser473, cat# 4060), p-Akt (Thr308, Cat# 13038), p-ERK1/2 (Thr202/Tyr204, cat# 9101), p-JNK (Thr183/Tyr185 cat# 9251), total-Akt (t-Akt, cat# 9272), t-ERK1/2 (Cat# 9102), t-JNK (Cat# 9258), mouse monoclonal antibody against glial fibrillary acidic protein (GFAP, cat# 3670) and U0126 were purchased from Cell Signaling (Beverly, MA).

### Cell culture

All animal experiments were approved by the Institutional Animal Care and Use Committee of Morehouse School of Medicine. Primary cortical astrocytes were isolated and cultured as described in a previous study with a few modification [30]. Briefly, mouse cerebral cortices were isolated from about E18 embryos and digested with 0.25% trypsin-EDTA for 10 min at 37°C. The cells were dissociated by gentle pipetting with glass pipette and cultured in T75 flask with dulbecco's modified eagle medium (DMEM) plus 10% FBS, 40 U/mL penicillin and 100 μg/mL streptomycin. Neurons, oligodendrocytes, and microglial cells were removed by shaking the flask at 180 RPM for 16–18h and washing with PBS twice. The purity of astrocytes was verified by staining with GFAP antibody. Cultures with > 95% GFAP positive cells were used for the experiments.

Human embryonic kidney (HEK293) cell line with inducible expression of human TRPM7 channels (HEK/M7) was a gift from Dr. A. Scharenberg (University of Washington). Cells were cultured in minimal essential medium (MEM) complemented with 10% FBS and antibiotics [31]. TRPM7 expression was induced by the addition of 1 μg/mL tetracycline.

### RNA interference

Knockdown of TRPM7 experiments were performed as described previously [28]. Briefly, siRNA against TRPM7 corresponding to coding region 5152–5172 (TRPM7 siRNA-1, Genebank# AY032951) [18] and 1630–1650 (TRPM7 siRNA-2, Genebank# NM_021450) [32] were synthesized by Invitrogen. Cells were transfected with 50 nM siRNA using transfection reagent lipofectamine RNAiMAX or lipofectamine 2000 (Invitrogen, Carlsbad, CA) according to the manufacturer’s instructions. Non-targeting siRNA (Invitrogen, Carlsbad, CA) was used as a control siRNA.

### RT-PCR and quantitative RT-PCR

RT-PCR was performed as described previously [28]. Briefly, total RNAs of astrocytes were extracted with RNA purification kit (Qiagen, Valencia, CA) and transcribed to cDNA using superscript First-strand synthesis system (Invitrogen, Carlsbad, CA). Quantitative RT-PCR was performed using SYBR Green supermix (Bio-rad, Richmond, CA) in C1000 Thermal cycler (Bio-rad) as described. The primer sequences were described in the “Table 1”.

**Table 1:** Primer sets sequence.

	**Targets**		**Primers sequence**	**Predicted size (bp)**
RT-PCR	TRPM7	S	5’-GATTTGCCCGTGATACCC-3’	493
		AS	5’-TTTCTGCTTGCACCGAGT-3’	
	β-actin	S	5’-CTGTCCCTGTATGCCTCTG-3’	218
		AS	5’-ATGTCACGCACGATTTCC-3’	
	TRPM4	S	5’-CCCTGAGGATGGTGTTGAGT-3’	176
		AS	5’-AGGAGCACTGGGATGTCAAT-3’	
	TRPM6	S	5’-CCAGGTGCCGGTAATAACA-3’	219
		AS	5’-CTCTTGTGGCTGCCTTAGGT-3’	
Quantitative RT-PCR	TRPM7	S	5’-TTTGGTGTTCCCAGAAAAGC-3’	175
		AS	5’-ACCAAGTTCCAGGACCACAG-3’	
	β-actin	S	5’- AGCCATGTACGTAGCCATCC-3’	228
		AS	5’- CTCTCAGCTGTGGTGGTGAA-3’	

S, indicated sense; AS, antisense.

### Electrophysiology

Whole-cell voltage-clamp recordings were performed as described previously [28]. Patch electrodes were constructed from thin-walled borosilicate glass (WPI) and had resistances of 1 to 3 MΩ. Currents were recorded using Axopatch 200B amplifier with pCLAMP software (Axon Instruments). They were filtered at 2 kHz and digitized at 5 kHz using Digidata 1322A. Data were eliminated from statistical analysis when access resistance was >10 MΩ or leak current was >100 pA at −60 mV. A multibarrel perfusion system was used to achieve a rapid exchange of external solutions. Standard extracellular solution contained (in millimolar) 140 NaCl, 5.4 KCl, 2 CaCl_2_, 1 MgCl_2_, 20 HEPES, 10 glucose (pH 7.4 adjusted with NaOH; 320–335 mOsm). Patch electrodes contained (in millimolar) 140 CsF, 10 HEPES, 1 CaCl_2_, 11 EGTA, 2 TEA, (pH 7.25 adjusted with CsOH, 290–300 mOsm). In order to potentiate the TRPM7-like current, the extracellular CaCl2 and MgCl2 were removed. For investigating the current–voltage (I–V) relationship, a voltage ramp between −100 mV and +100 mV from a holding potential of −60 mV was applied as described previously [33]. All experiments were done at the room temperature.

### Cell proliferation assay

Total LDH assay has been used to assess the cell proliferation as previously described [28, 34]. Cells were plated in 24-well plate at a density of 2–3 × 10^4^ cells/well. After starved for 24h in serum free DMEM, cells were transfected with siRNA or treated with indicated drugs for 3–4 days. Cells were washed twice with PBS and lysed with 1% Triton X-100 in PBS. Supernatant was taken for LDH assay according the manufacturer’s protocol of kit (Roche Diagnostics). The absorbance was examined by spectrometer (SpectraMax Plus, Molecular devices).

### Migration assay

Cells were starved with serum-free medium for 24h and the cell monolayer was scratched with a 1000 μl pipette tip. After washed 3 times with serum-free medium, the images of original wound area were taken with microscope (Olympus FSX100, 4.2×). Cells were incubated with DMEM containing 1% FBS for 3 days at 37°C. 3 days later, images were taken from the same location of wound area with the same microscope and analyzed using NIH Image J software. Migration rate was calculated as: % wound closure = [(Area of original wound—Area of wound after healing)/Area of original wound] × 100%.

### Immunostaining

Cells growing on coverslip pre-coated with Poly L-ornithine were washed twice with PBS and then fixed with 4% paraformaldehyde for 30 min. The cells were permeabilized with 0.2% Triton X-100 in PBS for 10 min and blocked with 3% BSA in PBS for 60 min. After incubated with primary antibody against TRPM7 (1:100) and GFAP (1:250) at 4°C overnight. The coverslips were washed three times at a 5 min interval and then incubated with FITC-conjugated goat anti-mouse secondary antibody for 1h at room temperature, mounted with DAPI solution followed by observing under fluorescence microscope.

### Western blotting

Western blotting was performed as described previously [28]. Briefly, cells were first lysed in RIPA buffer (50 mM Tris-HCL, pH 7.4, 150 mM NaCl, 1% Triton X-100, 0.5% Sodium deoxycholate, 0.1% SDS, protease inhibitor and phosphatase inhibitor cocktail). After centrifugation at 13,000g at 4°C for 10 min, the supernatant was collected. The aliquots were mixed with laemmli sample buffer and boiled at 95°C for 10 min. Proteins were separated by 10% SDS-PAGE, transfered to PVDF membranes, and then incubated with different primary antibodies against TRPM7 (1:200), β-actin (1:2000), p-p38 (1:1000), p-ERK1/2 (1:1000), p-JNK (1:500), p-Akt (Ser473, 1:1000), p-Akt (Thr308, 1:1000), t-p38 (1:500), t-ERK1/2 (1:1000), and t-JNK (1:500). Membranes were incubated with HRP or IRDye 800-conjugated secondary antibodies (1:2000) for 1h at room temperature. The signals were visualized with ECL kit or LI-COR Odyssey. The intensity of the protein band was quantified with Image J software (NIH).

### Mg^2+^ and Ca^2+^ assay

Intracellular Mg^2+^ concentration was quantified by QuantiChrom Magnesium Assay Kit (BioAssay Systems) according to the manufacturer’s instruction. Briefly, cells were washed 3 times with Ca^2+^ and Mg^2+^ -free PBS and collected in 100ul Ca^2+^ and Mg^2+^ -free PBS with a scraper. Suspension (10 μl) was taken for protein assay using BCA assay kit (Pierce). The remaining suspension was incubated with 5% (w/v) trichloroacetic acid at room temperature for 5 min. The supernatant was harvested by centrifuge at 14,000 rpm for 2 min, and some aliquots were taken for Mg^2+^ concentration assay according to the kit instruction. While intracellular Ca^2+^ levels were determined with the Ca^2+^-sensitive fluorochrome Fluo-3/acetoxymethyl ester (Fluo-3/AM) by a Becton Dickinson FACS Calibur flow cytometer as described in a previous study [35]. Briefly, cells were suspend in Tyrode’s buffer (134 mM NaCl, 12 mM NaHCO_3_, 2.9 mM KCl, 0.34 mM Na_2_HPO_4_, 1 mM MgCl_2_, 1mM CaCl_2_, 10 mM Hepes, pH 7.4), and incubated with 5 μM Fluo-3/AM at room temperature for 30 min in the dark. Extracellular free dye was washed away with Tyrode’s buffer. The fluorescence of Fluo-3 in cells was analyzed by flow cytometry (BD Bioscience).

### Statistical analysis

All data were presented as means ± SEM. Statistical analyses were done by Student *t* test. All the experiments were repeated at least 3 times. The differences were considered statistically significant when *p* < 0.05.

## Results

### Expression of TRPM7 in cortical astrocytes

**Fig 1:**
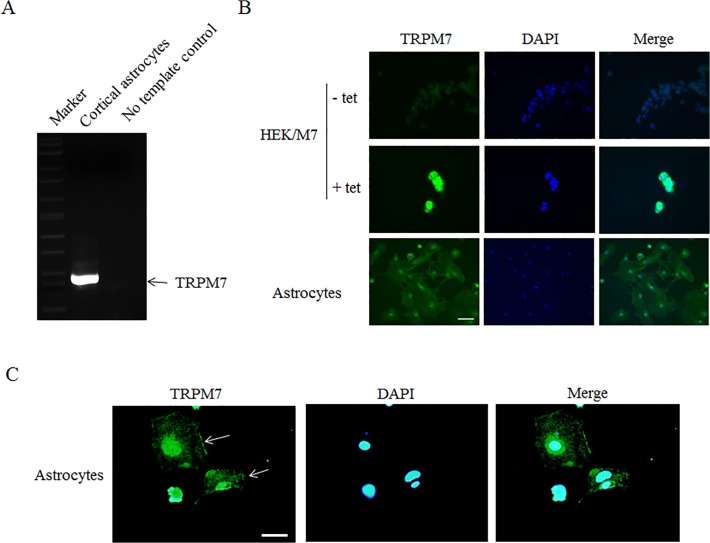
TRPM7 is expressed in mouse cortical astrocytes. **A**. Reverse transcript (RT)-PCR shows a clear band with the expected size exists in cortical astrocytes (left panel) but no band in no template control (right panel). **B**. Immunostaining shows that tetracycline (Tet) drastically increases TRPM7 expression (green) in TRPM7-expressing HEK293 cells (HEK/M7) compared to cells not treated with tetracycline (upper row). Cells are stained with DAPI (blue) to reveal the nuclei. Scale bar, 50 μm. C. Representative images show the localization of TRPM7 in astrocytes. Arrows indicate the cell membrane. Scale bar, 50 μm.

Cortical astrocytes were isolated from mouse cerebral cortex and purified as described in previous study [30]. Glial fibrillary acidic protein (GFAP, a marker of astrocytes) staining suggested that >95% of cells were astrocytes (S1 Fig.). Total RNAs were extracted from cortical astrocytes and reverse transcribed to cDNA. RT-PCR results showed a clear band of expected size in the astrocytes panel, but no band in the no-template control panel (Fig. 1A). Next, we examined the presence of TRPM7 protein by immunocytochemistry. As shown in Fig. 1B, tetracycline (Tet) drastically increased TRPM7 expression (green) in HEK293 cells with inducible expression of TRPM7 (HEK/M7) compared to cells not treated with tetracycline (Fig. 1B, upper row). Astrocytes showed a clear fluorescence signal when using the same antibody against TRPM7 under the same condition (Fig. 1B, under row). As shown in Fig. 1C, TRPM7 channels are located in whole cell body of astrocytes, and enriched in plasma membranes and nucleus (Fig. 1C). Taken together, these results indicate that TRPM7 channel is expressed in cultured mouse cortical astrocytes.

### Knockdown of TRPM7 in cortical astrocytes

**Fig 2:**
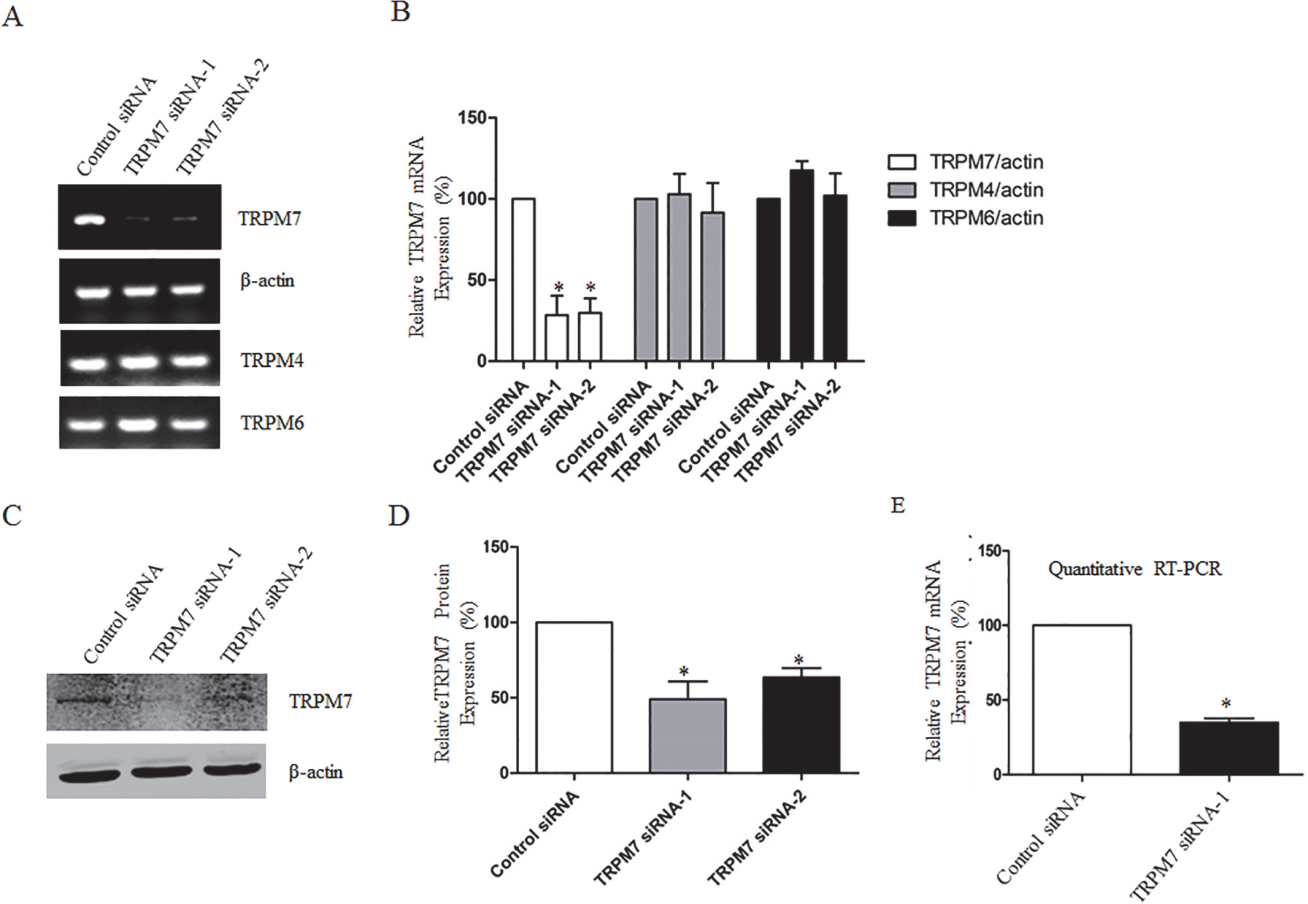
TRPM7 is down-regulated in mouse cortical astrocytes by siRNA. **A**. RT-PCR shows that transfection of astrocytes with specific TRPM7 siRNA decreases the mRNA level of TRPM7 compared to those with control siRNA. However, the mRNA levels of homologous genes TRPM4 and TRPM6 were not changed. **B**. Quantification of the expression of TRPM7, TRPM4 and TRPM6 mRNA in astrocytes treated with control siRNA or TRPM7 siRNA. Normalized TRPM7 mRNA expression shows a significant decrease after transfection with TRPM7 siRNA-1 (by 71.6% ± 11.9%) and siRNA-2 (by 70.2% ± 8.9%) as compared to control siRNA (*, *p*<0.05, n = 3). TRPM4 and TRPM6 mRNA levels were not changed (n = 3). **C**. Western blotting shows that TRPM7 siRNA-1 and siRNA-2 decrease the protein level of TRPM7 in astrocytes as compared to control siRNA. **D**. Quantification of TRPM7 protein level in astrocytes transfected with control siRNA and TRPM7 siRNAs. TRPM7 siRNA-1 significantly decreases TRPM7 protein expression by 50.9% ± 11.7%, while TRPM7 siRNA-2 decreases TRPM7 protein by 36.4% ± 6.1% (*, *p* < 0.05, n = 3). E. Quantitative RT-PCR confirms the reduction of TRPM7 mRNA normalized to β-actin in astrocytes transfected with TRPM7 siRNA-1 compared to those with control siRNA (*, p < 0.05, n = 3).

In order to further investigate the expression and role of TRPM7 in mouse cortical astrocytes, we synthesized two pairs of specific siRNAs against mouse TRPM7 as described in previous studies (see methods). As shown in Fig. 2A, TRPM7 siRNA-1 and siRNA-2 decreased the expression of TRPM7 mRNA by 71.6% ± 11.9% and 70.2% ± 8.9% respectively compared to the control siRNA (Fig. 2A and 2B). However, they had no effect on the mRNA expression of TRPM4 and TRPM6 (homologous genes for TRPM7). Consistently, western blotting analysis showed that TRPM7 siRNA-1 and siRNA-2 reduced the expression of TRPM7 protein by 50.9% ± 11.7% and 36.4% ± 6.1% respectively (Fig. 2C and 2D). In addition, quantitative RT-PCR demonstrated that the mRNA of TRPM7 in astrocytes was decreased to 34.9% ± 2.7% by TRPM7 siRNA-1 (Fig. 2E). We selected the TRPM7 siRNA-1 for the rest of study because of its higher efficiency.

### TRPM7-like currents in cultured astrocytes

**Fig 3:**
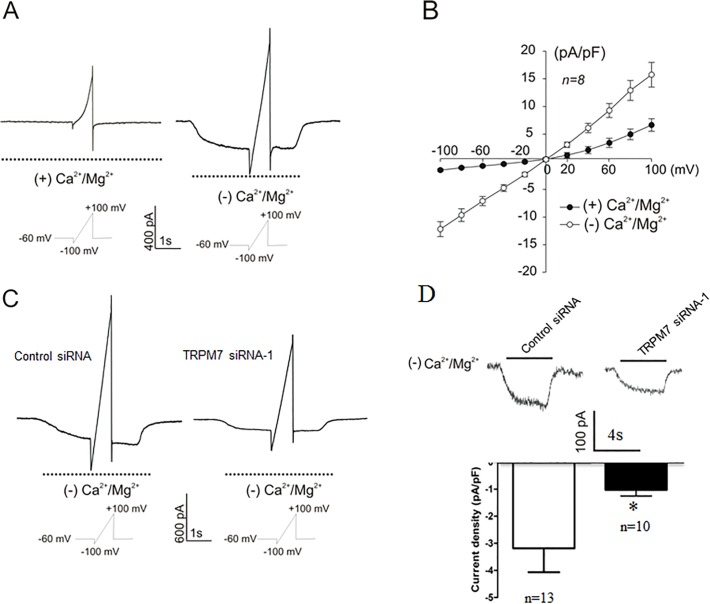
Functional TRPM7 channel is expressed in mouse cortical astrocytes. A. Voltage ramp (-100 to +100 mV) was applied for 4s from a holding potential of −60 mV. TRPM7 currents were potentiated in the absence of Ca^2+^ and Mg^2+^. B. Current-voltage relationship (I-V curve) was derived from A. n = 8. C. Representative traces showing the I-V curve of TRPM7 channels in astrocytes treated with control-siRNA or TRPM7-siRNA in the absence of Ca^2+^ and Mg^2+^. D. Removal of extracellular Ca^2+^ and Mg^2+^ potentiates the whole-cell TRPM7-like inward current at the −60 mV. Knockdown of TRPM7 reduces the peak current in astrocytes when transfected with TRPM7 siRNA. Quantification of current density shows that transfection of astrocytes with TRPM7 siRNA-1 (n = 10) significantly decreases the current density by 67.9% compared to control siRNA (n = 13, *, p < 0.05).

Next, we investigated the existence of functional TRPM7 channels in cultured astrocytes by whole-cell patch-clamp recording. As expected, removal of extracellular Ca^2+^ and Mg^2+^ potentiated the TRPM7-like inward currents in cortical astrocytes (n = 8, Fig. 3A and 3B). TRPM7 channels exhibit slightly outwardly-rectifying I-V relationship in the absence of divalent cations (Fig. 3B). Silencing TRPM7 with siRNA significantly decreased the current density by 67.9% (from −3.18 ± 0.88 pA/pF to −1.02 ± 0.23 pA/pF) (Fig. 3C and 3D), further confirming the expression of functional TRPM7 channels in cortical astrocytes.

### Effect of TRPM7 on the growth/proliferation of cortical astrocytes

**Fig 4:**
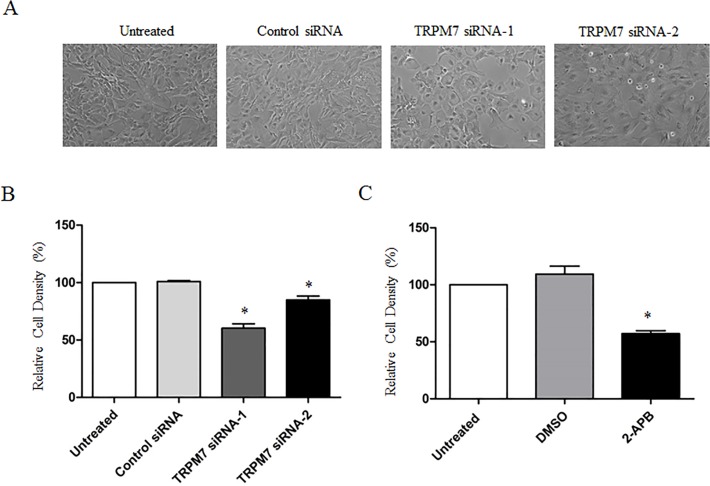
Silencing or suppression of TRPM7 inhibits cortical astrocyte proliferation. **A**. Representative images show the proliferation of astrocytes treated with control siRNA or TRPM7 siRNA. Scale bar: 50 μm. **B**. Quantification of the proliferation of astrocytes with different treatments. Transfection of astrocytes with TRPM7 siRNA-1 significantly reduces the proliferation by 40.2% ± 3.9% (n = 5; *, *p* < 0.05), while transfection of astrocytes with TRPM7 siRNA-2 reduces the proliferation by 15.9% ± 3.5% (n = 3) compared to control siRNA. Control siRNA has no effect on astrocyte proliferation compared to untreated control. **C**. TRPM7 inhibitor 2-APB (100 μM, n = 4) significantly decreases the proliferation of cortical astrocytes by 46.1% ± 2.5% (*, *p* < 0.05).

TRPM7 has been reported to affect the survival and proliferation of various cell types [15]. We first determined whether TRPM7 also influence astrocyte proliferation. As shown in Fig. 4A, the cell density of cortical astrocytes in culture wells transfected with TRPM7 siRNAs appears to be lower than those transfected with control siRNA (Fig. 4A). In order to quantify the change of cell number, we assayed total LDH value because the total amount of LDH is proportional to the total number of cells available in the culture [28, 34] (S2 Fig.). As shown in Fig. 4B, both TRPM7 siRNA-1 and siRNA-2 significantly inhibited the growth/proliferation of cortical astrocytes by 40.2% ± 3.9% and 15.9% ± 3.5% respectively (Fig. 4B). Similar to TRPM7 knockdown, a non-selective TRPM7 inhibitor 2-APB also inhibited the proliferation of cortical astrocytes by 46.1% ± 2.5% (Fig. 4C). In contrast, treatment of cells with vehicle DMSO had no effect compared to the non-treated cells (Fig. 4C). Since TRPM7 siRNA-1 seemed to be more effective than TRPM7 siRNA-2 in knocking down TRPM7 (Fig. 2) and astrocyte proliferation (Fig. 4B), we focused on TRPM7 siRNA-1 for the following experiments.

### Effect of TRPM7 on cortical astrocyte migration

**Fig 5:**
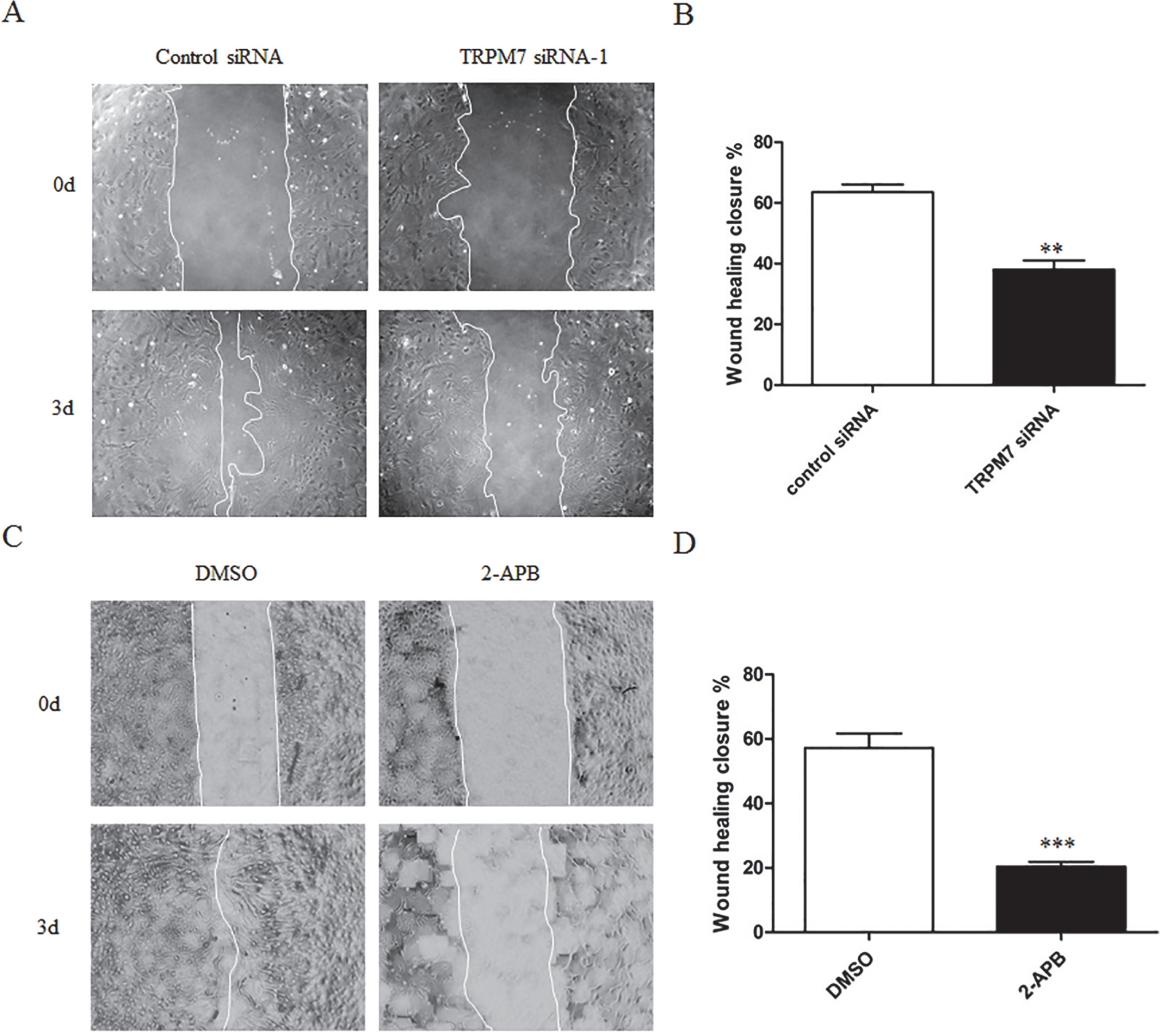
Silencing or suppression of TRPM7 inhibits cortical astrocyte migration. **A** and **C**. Representative images show wound healing closure of cortical astrocytes with different treatment after scratch injury from 0 day to 3 days. **B** and **D**. Quantification of wound healing closure of astrocytes. Silencing TRPM7 decreases the migration rate by 40.1 ± 11.5% (from 63.5% to 38.1%; **, *p*<0.01; n = 6) Inhibition of TRPM7 with 2-APB (100 μM) drastically impairs astrocyte migration by 64.2 ± 2.4% (from 57.2 ± 4.5% vs. 20.4 ± 1.4%; ***, *p* < 0.001; n = 6).

Astrocyte migration exerts a complex function in CNS lesion. Astrocytes could rapidly proliferate and migrate to the location of damage in response to brain injury [36, 37]. Thus, we next investigated the role of TRPM7 on astrocyte migration using the scratch wound healing model. Fig. 5A showed typical images of wound healing assay performed on HUVECs. Quantification analysis of these images showed that knocking down TRPM7 significantly inhibited astrocyte migration by 40.1% ± 11.5% compared to control siRNA (Control vs. TRPM7 siRNA-1: 63.5% ± 2.6% vs. 38.1% ± 23.0%, Fig. 5B). Consistently, 2-APB also inhibited astrocyte migration by 64.2% ± 2.4% compared to DMSO control (DMSO vs. 2-APB: 57.2% ± 4.5% vs. 20.4% ± 1.4%, Fig. 5C and 5D).

### Potential mechanisms underlying the effect of TRPM7-silencing on astrocyte proliferation and migration

**Fig 6:**
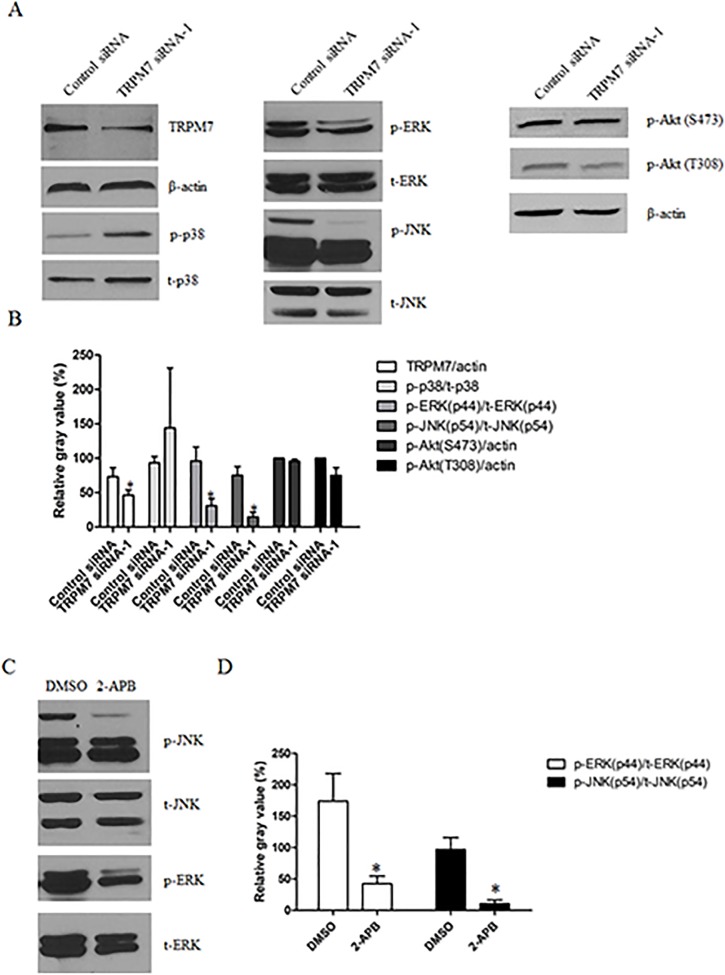
Silencing or suppression of TRPM7 impairs ERK and JNK signaling pathways. **A**. Western blot shows that TRPM7 siRNA decreases the expression of TRPM7 protein, phosphorylated ERK and JNK, but has no effect on phosphorylated p38 and Akt, as compared to the control siRNA. **B**. Densitometric analysis shows a decrease of p-ERK and p-JNK. TRPM7 siRNA significantly decreases the protein level of TRPM7 by 36.5% (n = 3), the phosphorylation of ERK and JNK by 67.8% ± 22.4% (n = 3) and 80.5% ± 25.3% (n = 3) respectively (*, *p* < 0.05). **C** and **D**. 2-APB significantly inhibits the phosphorylation of ERK and JNK by 75.6% ± 43.9% (n = 3) and 88.9% ± 19.1% (n = 4) respectively as compared to the DMSO control (*, *p* < 0.05).

**Fig 7:**
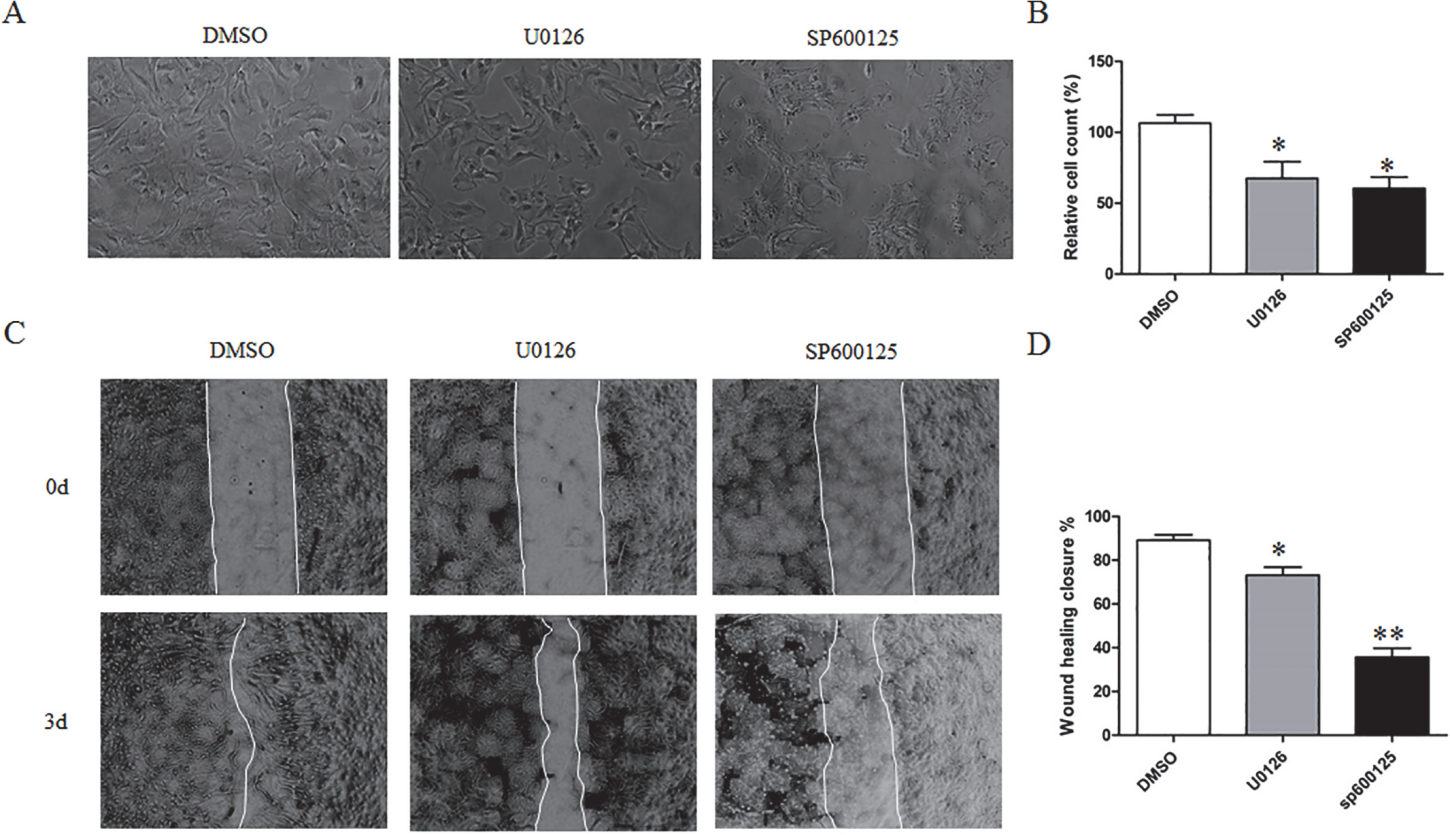
ERK and JNK inhibitors impair astrocyte proliferation and migration. **A** and **B**. MEK inhibitor (U0126, 10 μM) and JNK inhibitor (sp600125, 20 μM) inhibit astrocyte proliferation by 36.3% ± 11.8% and 42.9% ± 7.8% respectively (*, *p* < 0.05, n = 4). **C** and **D**. MEK inhibitor (U0126, 10 μM) and JNK inhibitor (sp600125, 20 μM) inhibit astrocyte migration by 17.9% ± 3.7% and 60.0% ± 4.1% respectively (*, *p* < 0.05; **, *p* < 0.01, n = 6).

MAPK and PI3K/Akt signaling pathways are involved in diverse cellular physiological processes including growth and proliferation. TRPM7 has been implicated in regulating MAPK and PI3K/Akt signaling in several cell types [27–29]. For these reasons, we investigated whether MAPK and PI3K/Akt signaling pathways are involved in TRPM7–mediated astrocyte proliferation and migration. To determine the activity of MAPKs and PI3K, we detected the phosphorylated form of ERK1/2, JNK, p38 and Akt using phospho-specific antibodies. Western blotting results showed that the phosphorylation of ERK and JNK in TRPM7-silent astrocytes clearly decreased as compared to control siRNA treated cells (Fig. 6A). Densitometric analysis of bands showed that p-ERK and p-JNK are reduced by 67.8% ± 22.4% and 80.5% ± 25.3% respectively (Fig. 6B). However, there are no significant changes of phosphorylation of p38 and Akt (Ser473 and Thr308) (Fig. 6A and 6B). Consistently, 2-APB inhibits the phosphorylation of ERK and JNK by 75.6% ± 43.9% and 88.9% ± 19.1% respectively (Fig. 6C and 6D). Furthermore, treatment of cortical astrocytes with U0126 (10 μM, MEK1/2 inhibitor) or SP600125 (20 μM, JNK inhibitor) inhibited cell proliferation by 36.3% ± 11.8% and 42.9% ± 7.8% respectively (Fig. 7A and 7B), and wound healing by 17.9% ± 3.7% and 60% ± 4.1% respectively (Fig. 7C and 7D). These results suggested that Silencing TRPM7 inhibited the proliferation and migration of cortical astrocytes likely through the reduced activity of ERK and JNK.

### Effect of TRPM7 on intracellular Mg^2+^ and Ca^2+^ concentration in astrocytes

**Fig 8:**
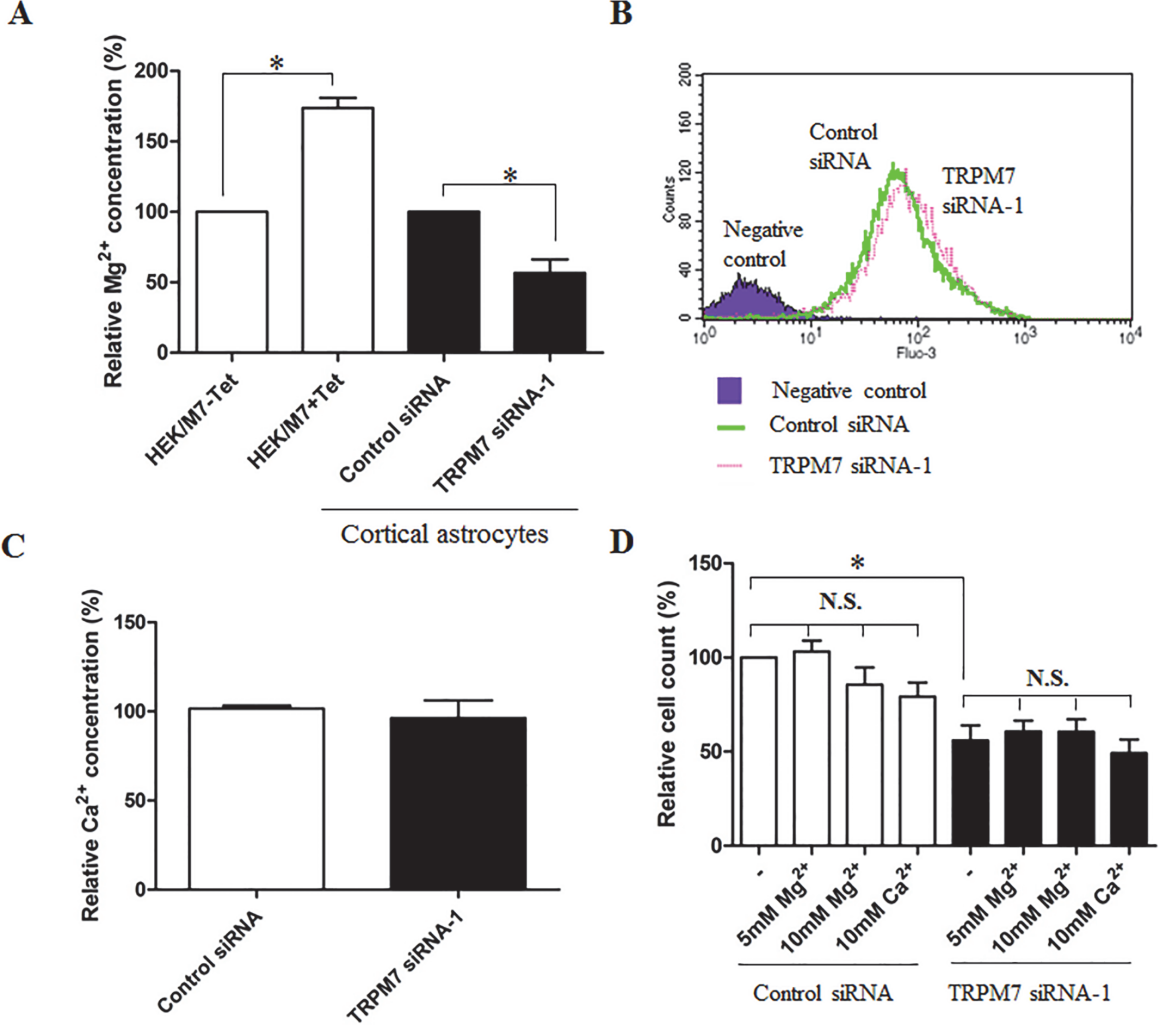
TRPM7 regulates intracellular Ca^2+^ and Mg^2+^ concentration of cortical astrocytes. **A**. Overexpression of TRPM7 in HEK/M7 cells induced by 1 μg/mL tetracycline drastically increases intracellular Mg^2+^ concentration by 73.7% ±7.23% (*, *p* < 0.05, n = 3). However, knockdown of TRPM7 in mouse cortical astrocytes significantly decreases intracellular Mg^2+^ by 43.5% ± 9.8% (*, *p* < 0.05, n = 4). **B**. Intracellular Ca^2+^ concentration of cortical astrocytes is analyzed by flow cytometry. All astrocytes loaded with Fluo-3 AM have a strong fluorescence signal compared to the negative control which has not been loaded with any dye. **C**. Quantification of intracellular Ca^2+^ concentration indicates significant difference between control siRNA and TRPM7 siRNA treatments (n = 5). **D.** increasing extracellular Ca^2+^ and Mg^2+^ has no effect on the proliferation of cortical astrocytes. Addition of Ca^2+^ and Mg^2+^ to growth medium has no effect on the proliferation of cortical astrocytes, and could not rescue the inhibition of astrocyte proliferation induced by silence of TRPM7 (*, *p* < 0.05, n = 4).

As a cation-permeable channel, TRPM7 was implicated in the regulation of Ca^2+^ and Mg^2+^ homeostasis in several kinds of cells [18, 38]. We next determined whether knocking down TRPM7 affected intracellular Mg^2+^ and Ca^2+^ concentration in cortical astrocytes. As shown in Fig. 8A, overexpressing TRPM7 in HEK293 cells dramatically increased intracellular Mg^2+^ by 73.7% ± 7.2%. In cortical astrocytes, knocking down TRPM7 significantly reduced the intracellular basal Mg^2+^ by 43.5% ± 9.8% (Fig. 8A). Flow cytometry analysis showed that most astrocytes loaded with Fluo-3 AM dye have a strong positive signal compared to negative control which did not load any dye (Fig. 8B). However, silencing TRPM7 did not change the basal level of intracellular Ca^2+^ in cortical astrocytes (Fig. 8C).

### Effect of extracellular Mg^2+^ and Ca^2+^ on the proliferation of astrocytes

Addition of extracellular cations has been shown to rescue the defect induced by TRPM7 silencing in several kinds of cells [38]. Using the proliferation assay, we determined whether extracellular Ca^2+^ and Mg^2+^ will rescue the defect of astrocytes transfected with TRPM7 siRNA. As shown in Fig. 8D, an addition of 5 mM—10 mM Mg^2+^ or 10 mM Ca^2+^ to the medium already contained 0.8 mM Mg^2+^ and 1.8 mM Ca^2+^ did not affect the growth/proliferation of astrocytes (Fig. 8D, left 4 white columns). Similarly, an addition of Mg^2+^ or Ca^2+^ also did not rescue the inhibited proliferation of TRPM7-silent astrocytes (Fig. 8D, right 4 black columns). These findings indicated that TRPM7 mediated astrocyte proliferation might be independent on the Ca^2+^ and Mg^2+^ influx. Alternatively, TRPM7 might not be the only pathway for Mg^2+^ entry in astrocytes. As was shown by Su et al., expression of a Mg^2+^ transporter could rescue the defects caused by knockdown of TRPM7 [39].

## Discussion

Astrocytes are specialized glial cells responsible for a wide variety of complex and essential functions in CNS, and play important roles in the regulation of disease processes. Several TRP channels have been reported to express in astrocytes and play crucial roles in astrocyte functions. Here we showed that functional TRPM7 channel was expressed in murine primary astrocytes. In addition, we showed that TRPM7 channel regulated astrocyte proliferation and migration through the ERK and JNK signaling pathways. As a Ca^2+^ and Mg^2+^-permeable channel, TRPM7 was found to regulate intracellular Mg^2+^ concentration of astrocytes. Together, our data reveal a new role for TRPM7 channel in the regulation of the physiological function of astrocytes in vitro.

TRPM7 is ubiquitously and highly expressed in brain and plays an important role in the pathophysiology of CNS like ischemic stroke [20, 21]. Most previous studies concentrated on the functions of TRPM7 channel in neurons under different pathophysiological status such as ischemic stroke, oxygen-glucose deprivation (OGD), oxidative stress, hypoxia and inflammation [18, 40]. In this study, we investigated the expression and function of TRPM7 in astrocyte, a specialized glia cell that outnumber neurons by over five fold. It is not surprising that functional TRPM7 channels were expressed in primary cultured cortical astrocytes, as is shown in other cells [28]. However, it is important that TRPM7 channel promotes astrocyte proliferation and migration via the ERK and JNK signaling pathways. TRPM7 channel plays a critical role in diverse cellular processes including proliferation, migration, adhesion, differentiation, and apoptosis. It is intriguing that TRPM7 channel performs different functions in different cells. TRPM7 promotes the proliferation, migration or differentiation in several types of cells such as macrophage [41], hepatic stellate cells [42], human prostate cells [43], human lung fibroblast [44], and pre-adipocytes [45]. In contrast, TRPM7 channel plays a negative function in human umbilical endothelial cells (HUVECs) including inhibition of proliferation and migration and enhancement of hyperglycemia-induced injury [28, 46, 47]. One possible reason for the different effects of TRPM7 is that there are significant differences in basal gene expression profiles and intracellular signaling machinery in different cells. Another possible reason may refer to the activity of TRPM7 itself because channel and kinase domain of TRPM7 can perform their functions independently. In addition, the substrates of TRPM7 kinase such as calpain, myosin heavy chain and annexin I might also be involved in these processes, which may result in different reaction [48–50]. Given that astrocytes exerts many essential complex functions in healthy CNS (see review [2]), TRPM7 channels may be a new potential regulator for astrocyte-related CNS physiological functions.

TRPM7 channel mediates several signaling pathways including MAPK and PI3K pathways which play important roles in cell proliferation and migration. For example, TRPM7 channel regulates PDGF-BB-induced proliferation of hepatic stellate cells via PI3K and ERK pathways [51]. Silencing TRPM7 promotes HUVEC proliferation by activating the ERK pathway [28]. TRPM7 mediates DT40 cell growth through the PI3K/Akt signaling pathway [29]. Notably, TRPM7 regulates cell proliferation or migration through different signaling pathways in different cells. In cortical astrocytes, knockdown of TRPM7 selectively impairs the activity of ERK and JNK, but has no effect on p38 and Akt. Furthermore, a variety of previous studies have also demonstrated that ERK and JNK played a crucial role in physiological functions of astrocytes including proliferation and migration [52, 53]. ERK was involved in both basal and stimulated astrocyte proliferation, while selective inhibitors such as PD98059 and U0126 led to the inhibition of astrocyte proliferation [52]. Similarly, JNK pathway was involved in astrocyte growth and proliferation as well. It has been shown that angiotensin II and III regulate astrocyte proliferation by activating JNK signaling [53, 54]. However, it does not exclude the possibility that other signal pathways like JAK/STAT pathway might also be involved in TRPM7-mediated astrocyte proliferation, just as TRPM7 channel regulates glioma stem cells through STAT3 and Notch signaling pathways [55]. Other ways might also be available because TRPM7 possesses both channel and kinase domain. In some case, silencing TRPM7 mimics the deficiency of magnesium by inhibiting cell proliferation and migration [38, 56]. In addition, the substrates of TRPM7 kinase like calpain, myosin heavy chain and annexin I might be involved in these processes as well [48]. However, we cannot exclude other potential factors which may also be involved in TRPM7-mediated astrocyte proliferation and migration. Additional experiments using gene chip microarray or proteomics to compare the TRPM7-silent and WT astrocytes may be helpful for investigating the detailed mechanisms.

As a Ca^2+^ and Mg^2+^-permeable channel, TRPM7 regulates intracellular Ca^2+^ and Mg^2+^ homeostasis in most cells. However, it does not mean that altering TRPM7 expression or activity could always produce a change in intracellular Ca^2+^ or Mg^2+^ concentration. For example, overexpression of TRPM7 in HEK293 cells does not cause Ca^2+^ overload [48], but increases Mg^2+^ concentration (Fig. 8A). Deletion of TRPM7 disrupts embryonic development and thymopoiesis without altering Mg^2+^ homeostasis [57]. In addition, Akita et al demonstrated that TRPM7 was not involved in bradykinin–induced Ca^2+^ influx in astrocytes [58]. Similarly, we found that silencing TRPM7 in astrocytes does not alter the concentration of intracellular Ca^2+^, but decreases the concentration of Mg^2+^. However, the exact mechanism by which TRPM7 selectively conducts Ca^2+^ or Mg^2+^ is unknown in these cells. Interestingly, Mg^2+^ plays an important role in the physiological functions of CNS [59]. Lowering the intracellular Mg^2+^, for example, is expected to compromise the integrity of blood-brain barrier (BBB) [59]. Magnesium has also been shown to prevent the loss of synapse and reverse cognitive deficits in Alzheimer’s disease [60]. In addition, Mg^2+^ can decrease brain edema formation after traumatic brain injury (TBI) by restoring the polarized state of astrocytes and by down-regulation of AQP4 channels in astrocytes [61]. It suggests that TRPM7-mediated change in Mg^2+^ concentration may play an important role in the pathophysiological processes of CNS. However, its exact roles still need further investigation. In some case, supplement of extracellular Mg^2+^ in growth medium could rescue the proliferative defect induced by TRPM7 deficiency [38, 62]. However, this did not occur in astrocytes in the present study (Fig. 8D). One possible reason is that astrocyte proliferation induced by TRPM7 silencing is independent of Mg^2+^ influx. Another possible reason is that it needs another Mg^2+^ transporter like SLC41A2 to rescue the deficiency [39]. Further studies are required to investigate these issues.

Altogether, our findings suggest an important role of TRPM7 and its potential mechanisms in astrocyte proliferation and migration, which might aid in the further understanding of astrocyte biology. Our current studies, however, have limitations including: 1) The lack of in vivo experimental evidence; 2) No highly selective TRPM7 inhibitors available; 3) Did not examine the potential role of TRPM7 in the pathological processes such as ischemia and oxidative stress.

## Supporting Information

S1 Fig Primary cortical astrocytes were isolated from mouse embryonic cortex, and cultured on coverslip pre-coated with poly-L-ornithine. Cells were incubated with GFAP primary antibody at 4°C overnight, and then incubated with FITC-conjugated second antibody at room temperature for 1h. Phase contrast image indicates total cells. Fluorescence image indicates GFAP positive astrocytes (green). No (-) primary antibody image indicates negative control.
(TIF)

S2 Fig Standard curve shows that LDH OD value is linearly proportional to astrocyte counts in a certain range (n = 5). (TIF)
