# Supplementary figures and images for: Silencing TRPM7 in Mouse Cortical Astrocytes Impairs Cell Proliferation and Migration via ERK and JNK Signaling Pathways
Source: PLoS One. 2015 Mar 23;10(3):e0119912. doi: 10.1371/journal.pone.0119912 (PMC4370640; doi:10.1371/journal.pone.0119912)

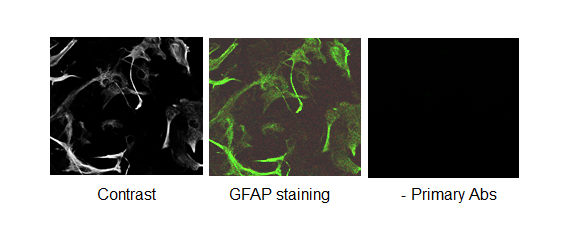

Supplement: S1 Fig — Cells were incubated with GFAP primary antibody at 4°C overnight, and then incubated with FITC-conjugated second antibody at room temperature for 1h. Phase contrast image indicates total cells. Fluorescence image indicates GFAP positive astrocytes (green). No (-) primary antibody image indicates negative control. (TIF) [file pone.0119912.s001.tif]

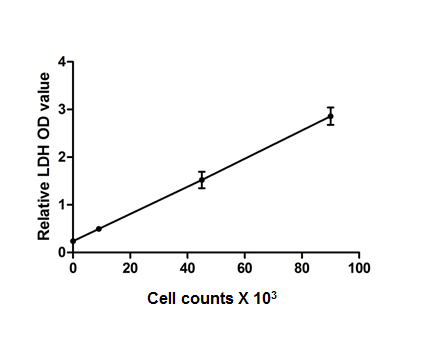

Supplement: S2 Fig — (TIF) [file pone.0119912.s002.tif]
